# A remarkable mixture of germanium with phosphorus and arsenic atoms making stable pentagonal hetero-prisms [M@Ge_5_E_5_]^+^, E = P, As and M = Fe, Ru, Os†

**DOI:** 10.1039/d0ra01316a

**Published:** 2020-05-27

**Authors:** Hung Tan Pham, Cam-Tu Dang Phan, Minh Tho Nguyen, Nguyen Minh Tam

**Affiliations:** Department of Chemistry, KU Leuven Celestijnenlaan 200F B-3001 Leuven Belgium; Laboratory of Computational Chemistry and Modelling, Quy Nhon University Quy Nhon Vietnam; Institute for Computational Science and Technology (ICST) Ho Chi Minh City Vietnam; Computational Chemistry Research Group, Ton Duc Thang University Ho Chi Minh City Vietnam nguyenminhtam@tdtu.edu.vn; Faculty of Applied Sciences, Ton Duc Thang University Ho Chi Minh City Vietnam

## Abstract

A pentagonal hetero-prismatic structural motif was found for singly transition metal doped M@Ge_5_E_5_^+^ clusters, where the transition metal atom is located at the centre of a (5/5) Ge_5_E_5_ prism in which Ge is mixed with either P or As atoms. Structural characterization indicates that each (5/5) Ge_5_E_5_ prism is established by joining of two Ge_3_E_2_ and Ge_2_E_3_ strings in a prismatic fashion rather than two Ge_5_ and E_5_ strings. Each string results from a remarkable mixture of Ge and E atoms and contains only one E–E connection due to the fact that Ge–E bonds are much stronger than E–E connections. From the donor–acceptor perspective, the Ge_5_E_5_ tube donates electrons to the M center, which behaves as an acceptor. NBO atomic charge and ELI_D analyses demonstrate such electrostatic interactions of the M dopant with a Ge_5_E_5_^+^ tube which likely induce thermodynamic stability for the resulting M@Ge_5_E_5_^+^ cluster. CMO analysis illustrates that the conventional 18 electron count is recovered in the M@Ge_5_E_5_^+^ cations.

## Abstract 2

Geometric shape of the lowest-energy structure M@Ge_5_E_5_^+^.

## Graphical abstract

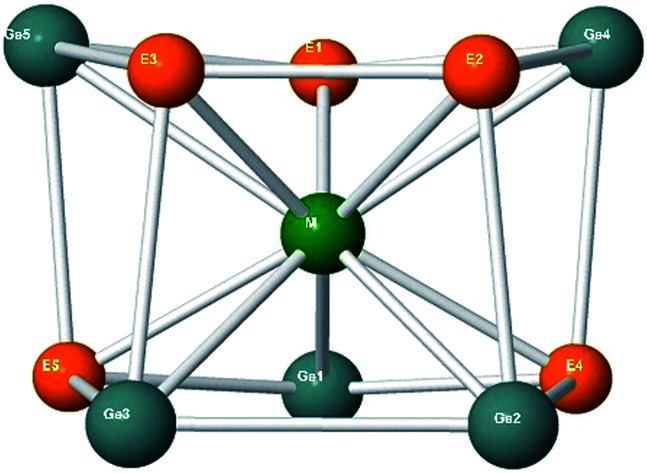

## 1. Introduction

Due to a potentially important role of germanium based compounds in semiconductors and optoelectronic industries,^1–4^ the geometric, electronic, thermodynamic and spectroscopic properties of small Ge clusters and their doped varieties have carefully been investigated by both theoretical and experimental methods alike.^5–15^ According to numerous previous studies on doped germanium clusters, singly transition metal doped germanium clusters provide us with a wide range of geometrical features. It is known that the M@Ge_16_ clusters with M = Ti, Zr, and Hf establish Frank–Kasper polyhedrons in which each metal dopant is encapsulated by a *T*_d_ Ge_16_ cage.^16^ Similar to silicon clusters, a hexagonal prism shape has been identified for the V@Ge_12_^−^, Mo@Ge_12_ and W@Ge_12_ clusters.^17–20^ Of the transition metal doped MGe_12_^−/0^ clusters, the gold doped AuGe_12_^−^ anion presents a high symmetry structure whose Au dopant is encapsulated by an *I*_h_ Ge_12_ host.^21^ Some previous theoretical studies found that the three-dimensional star-like structure can be constructed by the ionic interactions of seven satellite alkali cations with a flat E_5_^6^-pentagonal ring in which E is one of elements of group 14.^22–24^ Moreover, by using DFT calculations including van der Waals effects, Li *et al.* has point out the small Ge_6_, Ge_9_, and Ge_10_ clusters can play as the block units which can be connect together in order to form assembly materials and the van der Waals force impressively strengthens the covalent bond between different units, but plays less important role on the bonds in unit.^25^ Remarkably, it is highly particular that the pentagonal prism shape was experimentally observed for the CoGe_10_^3−^ and FeGe_10_^3−^ clusters in which either the Co or the Fe atom is centered in a *D*_5h_ (5/5) Ge_10_ pentagonal prism.^26,27^ A large number of systematic investigations were carried out to elucidate the structural evolution of singly metal doped germanium clusters at various charged states.^18–20,28–35^ Accordingly, an interplay between the metal dopant and the Ge-host gives rise to the richness on geometries varying from incomplete cage through encapsulated tube to Frank–Kasper polyhedron.

Within a great effort in the search for novel geometrical motifs of germanium-based cluster, multiple doping of P and As hetero-atoms to germanium hosts produced some symmetric hetero-fullerene structures. Following introduction of As atoms the mixed [V@Ge_8_As_4_]^3−^ and [Nb@Ge_8_As_6_]_3_^−^ hetero-fullerenes were generated. Structural identifications for the experimentally prepared [V@Ge_8_As_4_]^3−^ and [Nb@Ge_8_As_6_]^3−^ clusters showed that the V and Nb dopants are located at the central region of the Ge_8_As_4_ and Ge_8_As_6_ hetero-cages, respectively.^36,37^ Similarly, with dopant being Cr, Mo and W atoms, high symmetry structures were also observed in which the metal dopant is covered by a *D*_3h_ Ge_8_E_6_ frame with E = P and As. Subsequent theoretical studies pointed out that these M@Ge_8_E_6_ hetero-cages share an electron shell of [1S^2^1P^6^1D^10^1F^14^1G^18^2S^2^2P^6^2D^10^] enclosing 68 electrons. The existence of Ge-cages with mixed P and As elements suggests that a doping of P or As into a germanium host emerges as a good approach to generate high symmetry hetero-structures.

Although the MGe_10_*^q^* prismatic structures have gained so much attentions, their hetero-derivatives with P and As have been not considered yet. Indeed, while the FeGe_10_*^q^* was identified as a pentagonal prism in nine charge states with *q* being from −5 to +3, the isovalent RuGe_10_*^q^* clusters are of polyhedral geometry.^34,38^ Additionally, the compounds containing a P_5_ or As_5_ pentagon were found in the carbon-free as well as mixed M(Cp)E_5_ sandwich complexes. Within these coordination compounds, each P_5_ or As ring coordinates to a transition metal rather than forms any mixed-ring.^39–43^ It is subsequently predicted that M@Ge_5_P_5_^+^ and M@Ge_5_As_5_^+^ could be stable in a sandwich form where the M center is coordinated by both Ge_5_ and E_5_ rings. In this context, it is of interest to explore the effects of the P and As hetero-atoms to geometry of Fe@Ge_10_*^q^* pentagonal prism. With the aim to search for novel clusters possessing a stable tubular structural motif, we set out to carry out a theoretical investigation on geometries and electronic structure of the species Ge_5_E_5_^+^ in mixing the five germanium atoms with five P or As counterparts, and then they are singly doped by a transition metal (TM) giving rise to the doped M@Ge_5_E_5_^+^ clusters. The main role of the TM dopant is to stabilize the high symmetry tubular prism motif which is usually not stable in free forms. For a systematic exploration, we consider the elements of group 8 including Fe, Ru and Os as the dopant M. It turns out that such a mixture between Ge with either P or As leads to a set of remarkably stable pentagonal prisms containing an unprecedented combination of these elements.

## 2. Computational methods

In order to explore the potential energy surface (PES) of each of the M@Ge_5_E_5_^+^ systems considered, its guess geometries are generated by using a stochastic algorithm previously implemented by us.^44^ Our stochastic search method was improved based on the ‘random kick’ procedure reported by Saunders^45^ for exploring the low-lying isomers of compounds. According to this procedure, each atom of an initial structure is kicked to randomly move within a sphere of radius *r*, then the structures, generated from that, become the inputs for subsequent geometry optimizations using electronic structure calculations, and the “moving radius” *r* of atoms is the only variable controlled in this procedure. In our modified stochastic searching procedure, three additional variables will be controlled to provide better structures constructed for the following geometry optimizations. We modify this algorithm by adding a permutation subroutine in which each atom exchanges its position with all the others. For each MGe_5_E_5_^+^ system, we generate 1000 initial isomers for geometry optimization. This algorithm has been proven to be highly efficient in the search for the energetically lower-lying isomers of the systems containing various components.^46–48^ Additionally, on the basis of the well-known MGe_10_*^q^* structures that have already been reported in previous studies, we substitute Ge atoms by either P or As atoms, and thereby generate the initial isomers for the mixed M@Ge_5_E_5_^+^ systems.

All guessing structures of each series are geometrically optimized by using B3P86 functional in conjunction with small LANL2DZ basis set.^49^ Subsequently, the obtained structures, which have relative energy in range 50 kcal mol^−1^, will be selected to re-optimize using the same functional but in conjunction with a larger basis set, including the 6-311+G(d) set^50^ for Ge, P and As atoms, and the aug-cc-pVTZ or aug-cc-pVTZ-PP for Fe, Ru and Os^51,52^ in which PP stands for pseudo-potential. The current study utilizes the hybrid B3P86 functional due to it has previously been tested as suitable for treatment of geometrical and electronic structures of mixed clusters containing transition metals.^53^ All geometric optimizations and electronic structure calculations are performed using the Gaussian 09 suite of program.^54^ It should be noted that the cationic state is considered in order to probe the closed-shell electron configuration with a low spin state.

## 3. Result and discussion

### 3.1 Geometries

As for a convention, we label the structures considered as A.M.*x* in which A = P and As stand for Ge_5_P_5_ and Ge_5_As_5_ hosts, respectively, M = Fe, Ru and Os denotes the TM dopant, and finally, *x* = 1, 2, …, indicates the isomers with increasing relative energy. For the Ge_5_E_5_^+^ cations, the structures are denoted as A.*x*. Relative energies given here under are consistent with respect to the corresponding isomer *x* = 1.

**Fig. 1:**
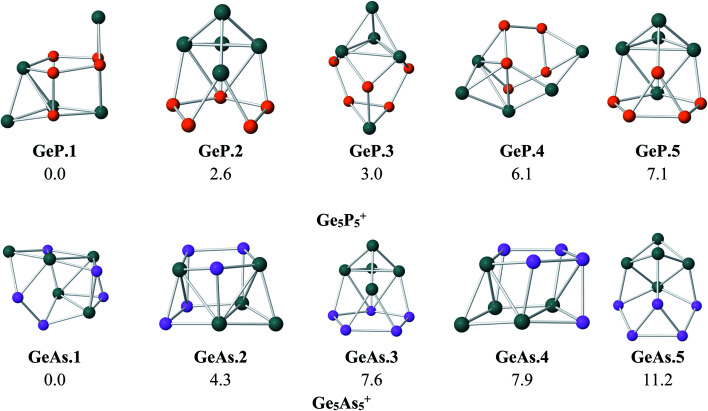
Shapes and relative energies (in kcal mol^−1^) of lower-lying isomers of Ge_5_P_5_^+^ and Ge_5_As_5_^+^ clusters. Geometry optimizations and energy calculations were performed at the B3P86/6-311+G(d) level.

To probe the effects of the metal dopant on the geometries of Ge_5_P_5_^+^ and Ge_5_As_5_^+^ cations, we first present in Fig. 1 the lower-lying isomers of both Ge_5_P_5_^+^ and Ge_5_As_5_^+^ cations obtained at the B3P86/6-311+G(d) level. No special shape is observed for both Ge–P and Ge–As mixed systems (Fig. 1). For Ge_5_P_5_^+^, the lowest energy structure P.1 contains four P–P connections, whereas P.2 turns out to contain a P_5_ cycle connected to a Ge_5_ counterpart and is only 3 kcal mol^−1^ higher in energy than P.1. The next isomers including P.3, P.4 and P.5 are significantly less stable. Regarding the Ge_5_As_5_^+^ cations, As.1 contains only one As–As bond but it emerges as the lowest-energy structure. The geometric characteristic of As.1 is completely different from that of the isovalent P.1. Remarkably, As.3 possesses an As_5_ pentagonal string and is 8 kcal mol^−1^ higher. Other higher energy isomers of the Ge_5_As_5_^+^ cation are also shown in Fig. 1.

**Fig. 2:**
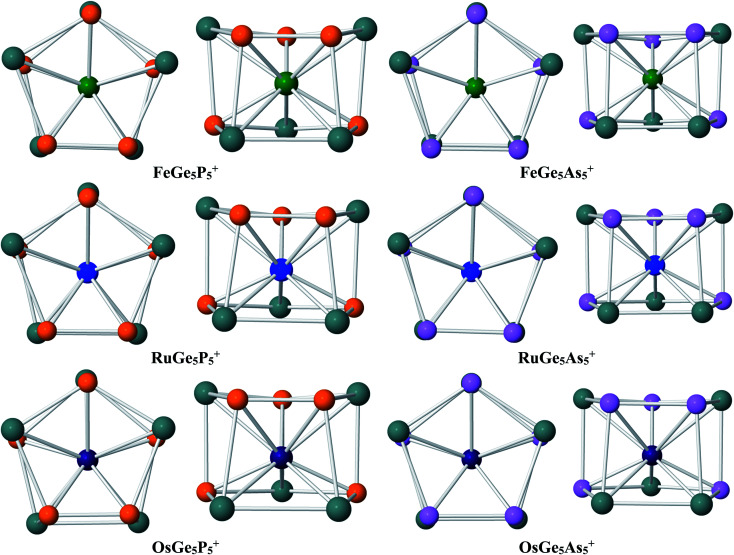
Shapes of the global energy minimum structures of M@Ge_5_E_5_^+^ with M = Fe, Ru and Os and E = P and As. Geometry optimizations were performed using the B3P86 functional with the 6-311+G(d) basis set for Ge and E and aug-cc-pVTZ basis set for Fe and aug-cc-pVTZ-PP basis set for Ru and Os metals.

Geometry identification for M@Ge_5_P_5_^+^ cations with M = Fe, Ru and Os clearly points out that a metal dopant M stabilizes the Ge_5_P_5_^+^ host into a double ring shape. The lower-lying isomers of M@Ge_5_P_5_^+^ clusters are displayed in Fig. 2, and also in Fig. S1–S3 of the ESI file.† Accordingly, the M@Ge_5_P_5_^+^ cations mainly feature a pentagonal prism, and each metal dopant, involving Fe, Ru and Os, is found to be located in the central region of a mixed (5/5) Ge_5_P_5_ double ring, which actually is formed by connecting Ge_4_P, Ge_3_P_2_, Ge_2_P_3_ and GeP_4_ pentagons together in a prismatic fashion. Of the latter, a combination of both Ge_3_P_2_ and Ge_2_P_3_ strings establishes the global energy minimum structure for the M@Ge_5_P_5_^+^ cation. No isomer having a (Ge_5_)M(E_5_)^+^ sandwich complex has been found. The appearance of M@Ge_5_P_5_^+^ double ring prism emphasizes the crucial role of the metal dopant Fe, Ru and Os in stabilizing a Ge_5_P_5_^+^ cation in a high symmetry form.

Similar to the Fe@Ge_10_^q^ cluster, a mixed Fe@Ge_5_P_5_^+^ cluster is thus stabilized in a pentagonal prism. Moreover, such a structural motif is consistently found for both Ru@Ge_5_P_5_^+^ and Os@Ge_5_P_5_^+^ as their ground state, whereas the Ru@Ge_10_*^q^* cluster does not exist. This result again demonstrates the important role of P atoms in formation of pentagonal prism, in such a way that a multiple doping of P atoms into a germanium host, or replacing of Ge by P atoms, appears to be an efficient approach to generate double ring structures for germanium-based clusters. It is interesting to note that the formation of P–P direct connections in each cluster series containing Fe, Ru and Os is in relation to the cluster stability. The most stable structure of Fe@Ge_5_P_5_^+^, Ru@Ge_5_P_5_^+^ and Os@Ge_5_P_5_^+^ cations contains each only one P–P bond, whereas other isomers having two or more P–P bonds are significantly less stable (Fig. S1–S3†). The isomers P.Fe.5, P.Fe.6 and P.Fe.7 contain each three P–P direct connections, and they are 10–15 kcal mol^−1^ higher in energy. Similarly, structures containing Ru and Os exhibit three or more P–P direct bonds are calculated to be highly unstable. Overall, introduction of Fe, Ru and Os dopants into a Ge_5_P_5_^+^ host establishes a (5/5) hetero-prism double ring structure for M@Ge_5_P_5_^+^ cations, but the Ge and P atoms are mixed in such a way that formation of two or more P–P direct bonds tend to destabilize the resulting clusters.

Regarding the M@Ge_5_As_5_^+^ clusters, a similar behavior is again observed. DFT calculations emphasize that a hetero-prismatic shape is again dominating as displayed in Fig. S4–S6 of the ESI file.† On the structural aspect, each of the Fe, Ru and Os dopants occupies a place of the central region of a prismatic cage formed by the Ge_4_As, Ge_3_As_2_, Ge_2_As_3_ and GeAs_4_ pentagons. Similar to M@Ge_5_P_5_^+^, disposition of both Ge_3_As_2_ and Ge_2_As_3_ pentagonal strings in an anti-prism form gives rise to the most stable structure for M@Ge_5_As_5_^+^, as depicted in Fig. 2. The similarity on geometric characteristic of M@Ge_5_As_5_^+^ clusters and their P homologues (M@Ge_5_P_5_^+^ cations) again emphasizes the crucial stabilizing role of Fe, Ru and Os metals in turning an irregular cage to a tubular structure. In case of FeGe_5_As_5_^+^, there is a competition for the ground state. Actually, the triplet As.Fe.1 and As.Fe.2 isomers, which are structures containing two and three As–As connections, are only ∼1 kcal mol^−1^ more stable than the singlet As.Fe.3, an isomer containing only one As–As bond. Additionally, the triplet ^3^A′′ (*C*_s_) As.Fe.3 is only ∼1 kcal mol^−1^ higher than its single state, so that they are competitive for ground state of the Fe@Ge_5_As_5_^+^ cluster. However, this result emphasizes that the existence of hetero-prism containing one As–As connection is a general tendency in M@Ge_5_As_5_^+^ clusters.

As in the P homologues, the thermodynamic stability of M@Ge_5_As_5_^+^ cations is found again in correlation with the number of direct As–As bonds. In fact, the isomer having one As–As connection is significantly more stable than those possessing two or more As–As bonds, as shown in Fig. S4–S6 of the ESI file.† In other words, formation of additional As–As bonds tends to destabilize the doped clusters.

**Fig. 3:**
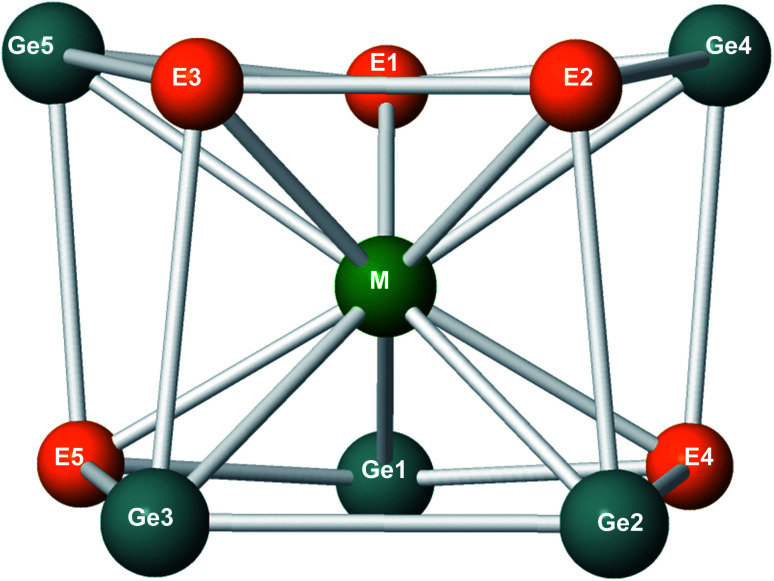
Geometric shapes of the lowest-energy structure of M@Ge_5_E_5_^+^.

The above structural identifications illustrate the coherent fact that the metal atoms of group 8 involving Fe, Ru and Os induce a great geometrical modification for the Ge_5_E_5_^+^ cations with E being an element in group of 15 (P and As). Both Ge_5_P_5_^+^P.1 and Ge_5_As_5_^+^As.1 cations do not exist in a special form, and more importantly, a prismatic shape is not observed at all for their lower-lying isomers. Incorporation of a metal of the group Fe, Ru and Os into such Ge_5_E_5_^+^ cations brings in a (5/5) pentagonal double ring prismatic shape for doped M@Ge_5_E_5_^+^ clusters, in which the strings are formed upon mixture of atoms. This appears to be a general tendency for this class of clusters (Fig. 3).

The geometric feature of M@Ge_5_E_5_^+^ clusters clearly shows that they prefer a mixed tubular shape rather than form a carbon-free sandwich complex. In fact, the existence of M@Ge_5_E_5_^+^ shows a different trend in which both P_5_ and As_5_ rings no longer exist. The sandwich structure (Ge_5_ME_5_)^+^ is extremely unstable, even it does not appear as a local minimum on the M@Ge_5_E_5_^+^ potential energy surface. It can thus be concluded that in the global minimum isomer of MGe_5_E_5_^+^, the metal center is coordinated by both Ge_3_E_2_ and Ge_2_E_3_ rings without any E_5_ string.

For a further characterization of the electron distribution, the bond length and Wiberg bond index (WBI) of the Ge–E, Ge–Ge and E–E bonds are tabulated in Table 2. For free M–E molecules, a bond length of ∼2.1 Å is found for M–P connections with M = Fe, Ru and Os, but their WBI values vary from 2.6, 3.2 to 3.5, respectively (Table 1). According to the usual meaning of WBI, P atom forms a triple bond with Fe and Ru while a nearly quadruple bond is identified for OsP dimer. A similar result is observed in M–As and M–Ge diatomic molecules where Os establishes a nearly quadruple bond with As and Ge, and a triple bond character is found for Fe–As, Fe–Ge, Ru–As and Ru–Ge dimeric species. The strength of M–E and M–Ge dimers tends to increase in going from M = Fe to Os. The WBI values of free Ge_2_, GeP and GeAs dimers are calculated to be 2.5, 2.7 and 3.1, respectively. As a consequence, they can be formally classified as a triple bond. Particularly, P_2_ and As_2_ have bond lengths of 1.9 and 2.2 Å, respectively, and the corresponding WBI values amount to 3.6 and 3.5.

**Table 1:** Geometrical parameters (distance in angstrom) and WBI (a. u.) of M@Ge_5_E_5_^+^ prisms

	Fe@Ge_5_P_5_^+^	Ru@Ge_5_P_5_^+^	Os@Ge_5_P_5_^+^		Fe@Ge_5_As_5_^+^	Ru@Ge_5_As_5_^+^	Fe@Ge_5_As_5_^+^
M–P1	2.3/1.2	2.4/1.6	2.5/1.5	M–As1	2.4/1.1	2.5/1.4	2.6/1.5
M–P2	2.3/1.2	2.4/1.4	2.4/1.6	M–As2	2.4/1.1	2.5/1.4	2.6/1.6
M–P4	2.3/1.3	2.3/1.2	2.3/1.9	M–As4	2.4/1.1	2.4/1.6	2.4/1.9
M–Ge4	2.6/1.0	2.7/1.2	2.7/1.4	M–Ge4	2.6/0.9	2.7/1.2	2.7/1.3
M–Ge1	2.5/1.1	2.6/1.3	2.6/1.5	M–Ge1	2.6/1.0	2.6/1.3	2.6/1.5
M–Ge2	2.5/1.1	2.6/1.4	2.5/1.6	M–Ge2	2.6/0.9	2.6/1.4	2.6/1.6
P2–P3	2.2/1.0	2.2/1.0	2.2/0.1	As2–As3	2.5/0.9	2.4/1.0	2.4/1.0
P1–Ge4	2.5/0.7	2.4/0.9	2.4/0.8	As1–Ge4	2.5/0.9	2.5/0.9	2.5/0.8
P2–Ge4	2.5/0.8	2.5/0.7	2.5/0.7	As2–Ge4	2.5/0.7	2.6/0.8	2.9/0.5
P1–Ge1	2.5/0.7	2.6/0.6	2.6/0.6	As1–Ge1	2.6/0.7	2.7/0.6	2.7/0.6
P2–Ge2	2.5/0.7	2.6/0.5	2.7/0.4	As2–Ge2	2.6/0.8	2.7/0.6	2.7/0.5
P4–Ge4	2.5/0.7	2.6/0.6	2.6/0.5	As4–Ge4	2.6/0.7	2.7/0.6	2.6/0.7
P4–Ge1	2.4/0.9	2.6/0.6	2.6/0.6	As4–Ge1	2.5/0.9	2.6/0.7	2.6/0.6
Ge3–Ge2	2.6/0.7	2.8/0.5	2.9/0.5	Ge3–Ge2	2.7/0.7	2.7/0.8	2.7/0.5

Within the M@Ge_5_P_5_^+^ clusters, P–P connections have bond length of ∼2.2 Å and WBI values of ∼1.0 clearly indicating a single bond character. A similar single bond character is found for Ge–Ge, which exhibits a WBI value of 0.7 in Fe@Ge_5_P_5_^+^, and 0.5 in both Ru@Ge_5_P_5_^+^ and Os@Ge_5_P_5_^+^. Connections of Ge with P atoms are characterized by WBI values in the range of 0.4–0.9, also implying a Ge–P single bond. Accordingly, Ge and P atoms form Ge–Ge, Ge–P and P–P single bonds in the Ge_5_P_5_ prismatic tube. A similar pattern is observed for the Ge_5_As_5_ cages in which Ge–As connections have WBI values of 0.5–0.9 for Ge–As bonds, and ∼1.0 for As–As and Ge–Ge bonds. It is important to explore the bonding between Ge_3_E_2_ and Ge_2_E_3_ rings. The connectivities associated with the superposition between both Ge_3_E_2_ and Ge_2_E_3_ strings is identified as single bond according to WBI results. Therefore, it is not possible to consider the pentagonal Ge_3_E_2_ and Ge_2_E_3_ rings of M@Ge_5_E_5_^+^ as two independent rings. On the other hand, the M@Ge_5_E_5_^+^ is a tubular cluster rather than a [(Ge_3_E_2_) M (Ge_2_E_3_)]^+^ carbon-free sandwich complex.

For connections containing the metal atom, the M–P bonds of M@Ge_5_P_5_^+^ are not only significantly longer than those of the corresponding M–P diatomic molecules, but their WBI values are also found to be in a range of 1.2–1.9. Similarly, M–As connections of M@Ge_5_As_5_^+^ prism have WBI values varying from 1.1 to 1.9. Hence, metal atoms form, in connecting with P and As atoms, stronger bonds than single bonds. In comparison to free MGe, MP and MAs dimers, formation of M@Ge_5_E_5_^+^ prisms significantly reduces the strength of the corresponding connection. As shown in Table 2, the M–Ge connections have WBI values of ∼1–2, whereas the WBI of free MGe species is greater than 2. In other words, the strength of a M–Ge bond is reduced when a doped M@Ge_5_E_5_^+^ prism is established. Overall, the WBI analysis indicates that Ge and E atoms either E = P or As, connect together by a single bond whereas a metal atom gives rise to multiple bonds in interacting with the Ge, P and As elements.

**Table 2:** Bond length (*d*, angstrom) and WBI (a.u.) of MGe, ME, E_2_ and GeE diatomic molecules

Molecule	*d*	WBI	Molecule	*d*	WBI	Molecule	*d*	WBI
Fe–P	2.1	2.6	Ru–P	2.1	3.2	Os–P	2.1	3.5
Fe–As	2.2	2.4	Ru–As	2.2	3.3	Os–As	2.1	4.1
Fe–Ge	2.3	2.3	Ru–Ge	2.2	3.3	Os–Ge	2.1	3.5
Ge–Ge	2.4	2.5	Ge–P	2.2	2.7	Ge–As	2.4	3.1
P–P	1.9	3.6	As–As	2.1	3.5			

Although both P_2_ and As_2_ dimeric molecules are highly stable, as indicated by large values of their dissociation energies and WBI, the appearance of P–P and As–As connections in a M@Ge_5_E_5_^+^ prismatic cluster tends to destabilize it. Within the most stable isomer of a M@Ge_5_E_5_^+^ cluster, both P–P and As–As connections are identified as single bonds, according to WBI results. Therefore, in order to rationalize the rather negative effect of the P–P and As–As connections on the stability of M@Ge_5_E_5_^+^ cluster, the dissociation energies (DE) of the E–E bonds as evaluated from the homolytic breaking H_2_E–EH_2_ → 2EH_2_, H_3_Ge–EH_2_ → Ge_3_H_3_ + EH_2_ and H_3_Ge–GeH_3_ → 2GeH_3_ processes which describe well the dissociation of E–E, Ge–E and Ge–Ge single bonds, are calculated and given in Table 1. Accordingly, the DEs of the H_3_Ge–GeH_3_ bond has a value of 66 kcal mol^−1^ whereas the DE values of H_3_Ge–PH_2_ and H_3_Ge–AsH_2_ are computed to be ∼60 kcal mol^−1^. The H_2_P–PH_2_ and H_2_As–AsH_2_ species have DEs of ∼50 kcal mol^−1^ which are significantly smaller than the others. This result points out both P–P and As–As single bonds are consistently weaker than the mixed Ge–P, Ge–As and the pure Ge–Ge counterparts. Therefore, formation of Ge–P, Ge–As and Ge–Ge bonds is expected to give raise more thermodynamic stability to M@Ge_5_E_5_^+^ prismatic structure than the P–P and As–As connections. As a result, the most stable isomer of each M@Ge_5_E_5_^+^ cluster contains only one P–P or As–As bond whereas eight Ge–E connections are formed (Table 3).

**Table 3:** Dissociation energy (DE, kcal mol^−1^) and bond length (*r*, Å) of H_2_E–XH_3_ molecules (TPSSh/6-311++G(d,p))

	DE (E–X)	*r* (E–X)
H_2_P–PH_2_	50.3	2.26
H_2_As–AsH_2_	47.0	2.47
H_3_Ge–GeH_3_	66.2	2.43
H_3_Ge–PH_2_	60.7	2.34
H_3_Ge–AsH_2_	57.9	2.44

### 3.2 Chemical bonding analysis: donor–acceptor complex

Interaction between a metal dopant M with a Ge_5_E_5_^+^ cage is further probed using the NBO atomic charges (Table 2) that illustrate that metal atoms bear a largely negative charge, ∼−3.5 electron. On the other hand, the Ge_5_E_5_^+^ prismatic double ring supplies electrons to the metal dopant, and thereby establishes a negatively charged center M^*δ*−^. Each M@Ge_5_E_5_^+^ prismatic structure can thus be regarded as a [M^*δ*−^ (Ge_5_E_5_)^*δ*+^]^+^ donor–acceptor complex where the metal atom M behaves as an acceptor centre and the Ge_5_E_5_^+^ cage plays as a donor moiety.

**Fig. 4:**
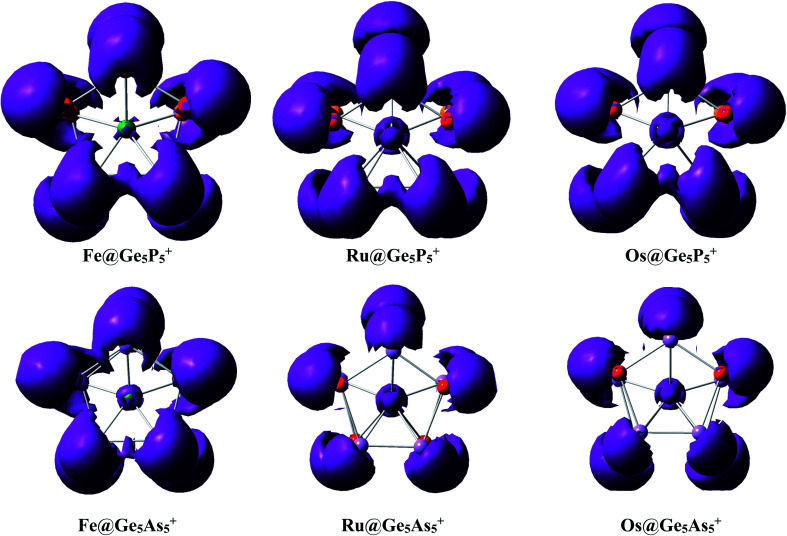
ELI_D maps plotted at the bifurcation value of 1.3 for M@Ge_5_E_5_^+^.

An analysis of the electron distribution using the electron localization indicator (ELI_D)^55^ is carried out to further explore the bonding phenomena of M@Ge_5_E_5_^+^ clusters. As shown in Fig. 4, at the bifurcation value of 1.3, a di-synaptic basin, *V*(E,E), is clearly observed for E–E connections in all clusters considered. Similarly, localization domains are found for either Ge–P or Ge–As connection indicating their covalent bond character. The *V*(M,Ge) and *V*(M,E) basins with M = Fe, Ru and Os, and E = P are found at lower bifurcation values suggesting that the connections containing metal atom have an ionic character. This result is consistent with the NBO analysis given above in which Ge_5_E_5_^+^ is shown to transfer much electron to the M center. Both NBO and ELI_D analyses illustrate that the electrostatic interaction of a negatively charged metal dopant with a Ge_5_E_5_^+^ positively charged tubular moiety contributes to a thermodynamic stabilization of the resulting M@Ge_5_E_5_^+^ hetero-prisms.

### 3.3 The 18 electron count

**Fig. 5:**
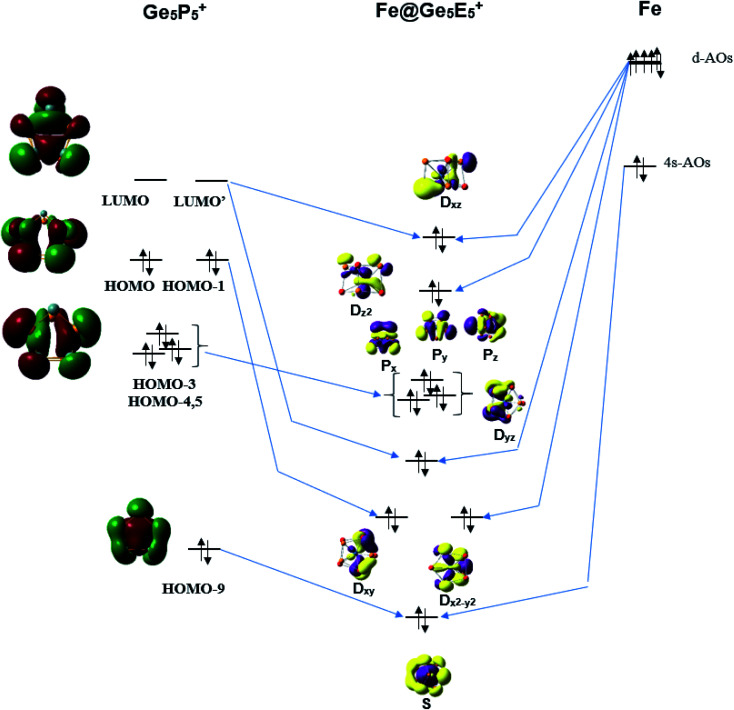
A representative orbital interaction diagram of Fe with Ge_5_P_5_^+^ prism producing MOs containing 18 electrons.

The stability of the singly metal doped tubes is often rationalized by using the classical electron count in which the metal atom receives electrons to gain a fulfilled d^10^ configuration. It should however be noted that the Fe, Ru and Os atoms have a general electron configuration of [*ns*^2^(*n* − 1)d^6^], which thus needs 4 additional electrons to fill their (*n* − 1)d^10^ shell. NBO analysis given above indicates that each Ge_5_E_5_^+^ cage transfers an amount of ∼3.5 electron to the metal center. In other words, each Ge_5_E_5_^+^ cage effectively provides 4 valence electrons to establish a closed (*n* − 1)d^10^ subshell for the metal M, and thereby stabilizes the M@Ge_5_E_5_^+^ tubular prism. Following this line of argument, the orbital interaction diagram between the Fe atom and a Ge_5_P_5_^+^ prism is constructed and displayed in Fig. 5. The result constructed for the Ge_5_P_5_^+^ case can certainly be generalized for the other derivatives.

According to Fig. 5, the 4s-AO of Fe atom involves interaction with the HOMO-9 of Ge_5_P_5_^+^ and produces an S level for Fe@Ge_5_P_5_^+^. The D_*xy*_ and D_*x*^2^–*y*^2^_ levels are produced upon interaction of the HOMO and HOMO-1 with 3d-AOs of Fe. Interaction of the LUMO and LUMO+1 of Ge_5_P_5_^+^ prism with 3d-AOs of Fe induces the D_*xz*_ and D_*yz*_ eigenstates of Fe@Ge_5_P_5_^+^. The 3d_*z*^2^_ AO of Fe becomes D_*z*^2^_ of Fe@Ge_5_P_5_^+^ prismatic cluster. The P_*x*_, P_*y*_ and P_*z*_ levels of Fe@Ge_5_P_5_^+^ are mainly contributed by the HOMO-3,4,5 of Ge_5_P_5_^+^ cage. Finally, orbital interactions of the Fe dopant with Ge_5_P_5_^+^ prism produce an electron configuration of [S^2^P^6^D^10^] which in fact contains 18 valence electrons.

A similar orbital interaction is also found for other clusters including RuGe_5_P_5_^+^, OsGe_5_P_5_^+^ and MGe_5_As_5_^+^ as shown in Fig. S7–S11 of ESI file.† These results show that metal dopants Fe, Ru and Os gain each 4 electrons from the Ge_5_E_5_^+^ cage to fulfill its (*n* − 1)d^10^ level, and thereby induce an enhanced stability for the M@Ge_5_E_5_^+^ prismatic structures, which not only share a general structural motif but also have a same stabilizing mechanism where the metal center gains 4 electrons from Ge_5_E_5_^+^ prismatic cage to establish a 18 electron configuration. It is interesting that the polyanions Co@Ge_10_^3−^ and Fe@Ge_10_^4−^ pentagonal prismatic clusters, which are iso-valent with Fe@Ge_5_E_5_^+^, are stabilized by a similar mechanism. They are similar to Ge_5_E_5_^+^ prisms; the Ge_10_ prismatic host also supplies electrons to fulfill 3d^10^ levels of Co and Fe centers, and subsequently establishes a 18 electron configuration.^38,56^ This gives an emphasis that hetero-atoms including P and As not only replace Ge position in a prismatic framework but also provide electrons to fulfil 18 electron configuration.

## 4. Concluding remarks

In summary, we presented a theoretical investigation on geometry, stability and chemical bonding of the Ge_5_E_5_^+^ and MGe_5_E_5_^+^ cationic clusters with E = P, As; M = Fe, Ru and Os. Structural identifications clearly pointed out that the doping of a transition metal atom greatly influences to geometry of the Ge_5_E_5_^+^ cage. Structurally, the singly doped M@Ge_5_E_5_^+^ clusters are stabilized in pentagonal hetero-prism shape whereas the Ge_5_E_5_^+^ cation is not in any special form. Each hetero-prismatic structure is formed by superposition of the Ge_4_E, Ge_3_E_2_, Ge_2_E_3_ and GeE_4_ pentagons together in prismatic fashion, but the combination of Ge_3_E_2_ and Ge_2_E_3_ strings peculiarly establishes the global minimum structure for M@Ge_5_E_5_^+^ cations. Interestingly, the cluster contains only one E–E connection exhibits the lowest-energy while a structure possesses two or more E–E bonds is significantly less stable. Ge–E bonds are in fact stronger than E–E connections. Within the donor–acceptor perspective, with acceptor being the metal dopant, the GeE cage donates around 4 electrons to the M center and then stabilize M@Ge_5_E_5_^+^ clusters. A CMO analysis illustrates that the conventional 18 electron count is effectively recovered in the stabilized M@Ge_5_E_5_^+^ cations.

## Conflicts of interest

The authors declare no competing financial interest.

## Supplementary Material

RA-010-D0RA01316A-s001
