# Supplementary material for: A remarkable mixture of germanium with phosphorus and arsenic atoms making stable pentagonal hetero-prisms [M@Ge5E5]+, E = P, As and M = Fe, Ru, Os
Source: RSC Adv. 2020 May 27;10(34):19781–9. doi: 10.1039/d0ra01316a (PMC9054236; doi:10.1039/d0ra01316a)
Supplement: RA-010-D0RA01316A-s001 [file RA-010-D0RA01316A-s001.pdf]

Electronic Supplementary Material (ESI) for RSC Advances.
This journal is © The Royal Society of Chemistry 2020

Remarkable Mixture of Germanium with Phosphorus and Arsenic Atoms Making Stable Pentagonal Hetero-Prisms $[M@Ge_5E_5]^+$, E = P, As and M = Fe, Ru, Os

Hung Tan Pham,[a,b,c] Cam-Tu Dang Phan,[d] Minh Tho Nguyen,[c,e] Nguyen Minh Tam[a,b,*]

[a] *Computational Chemistry Research Group, Ton Duc Thang University, Ho Chi Minh City, Vietnam*

[b] *Faculty of Applied Sciences, Ton Duc Thang University, Ho Chi Minh City, Vietnam*

[c] *Department of Chemistry, KU Leuven, Celestijnenlaan 200F, B-3001 Leuven, Belgium*

[d] *Laboratory of Computational Chemistry and Modelling, Faculty of Science, Quy Nhon University, Quy Nhon, Vietnam*

[e] *Institute for Computational Science and Technology (ICST), Ho Chi Minh City, Vietnam*

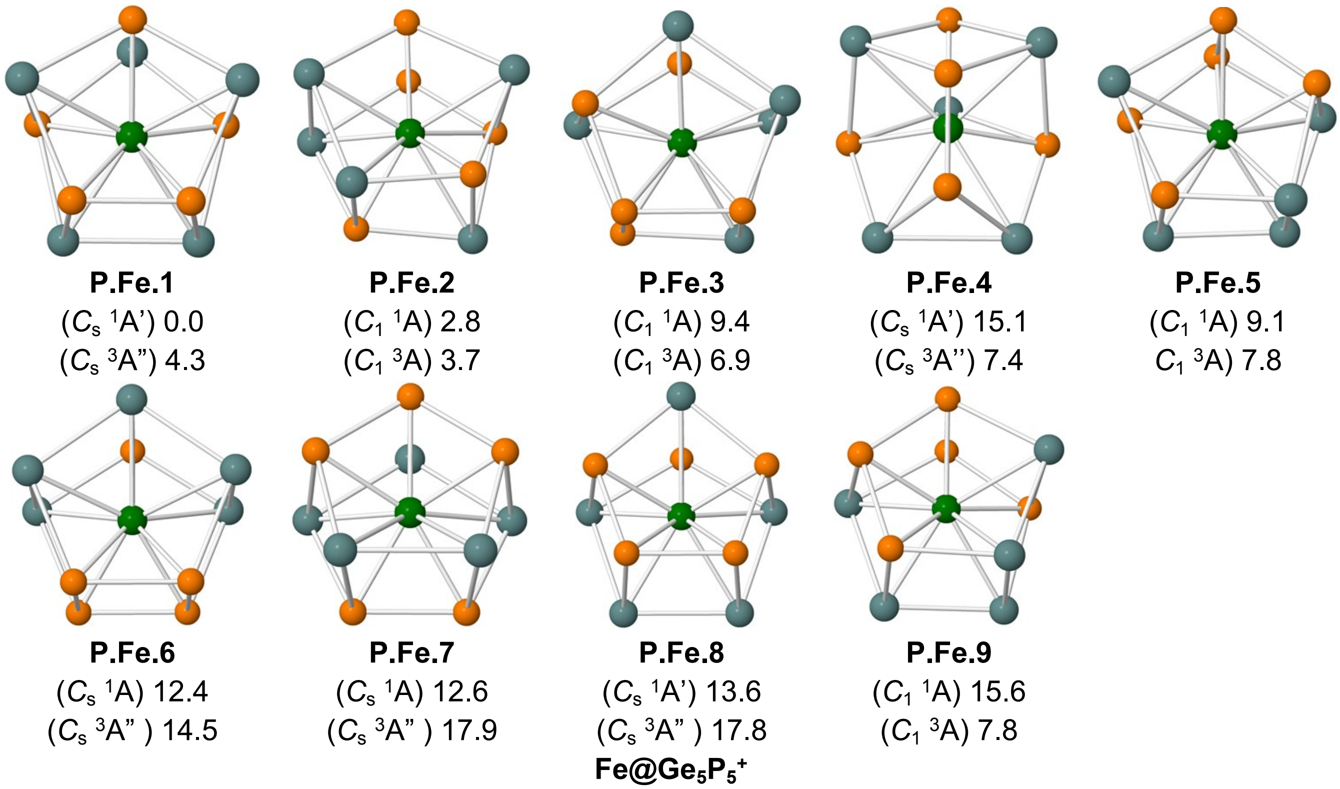

Figure S1. Shapes, and relative energies (in kcal/mol) of the lower-lying isomer of $FeGe_5P_5^+$ cluster. The geometry optimizations and energy calculations were performed using B3P86 functional with 6-311+G(d) basis set for Ge, P and aug-cc-pVTZ basis for Fe.

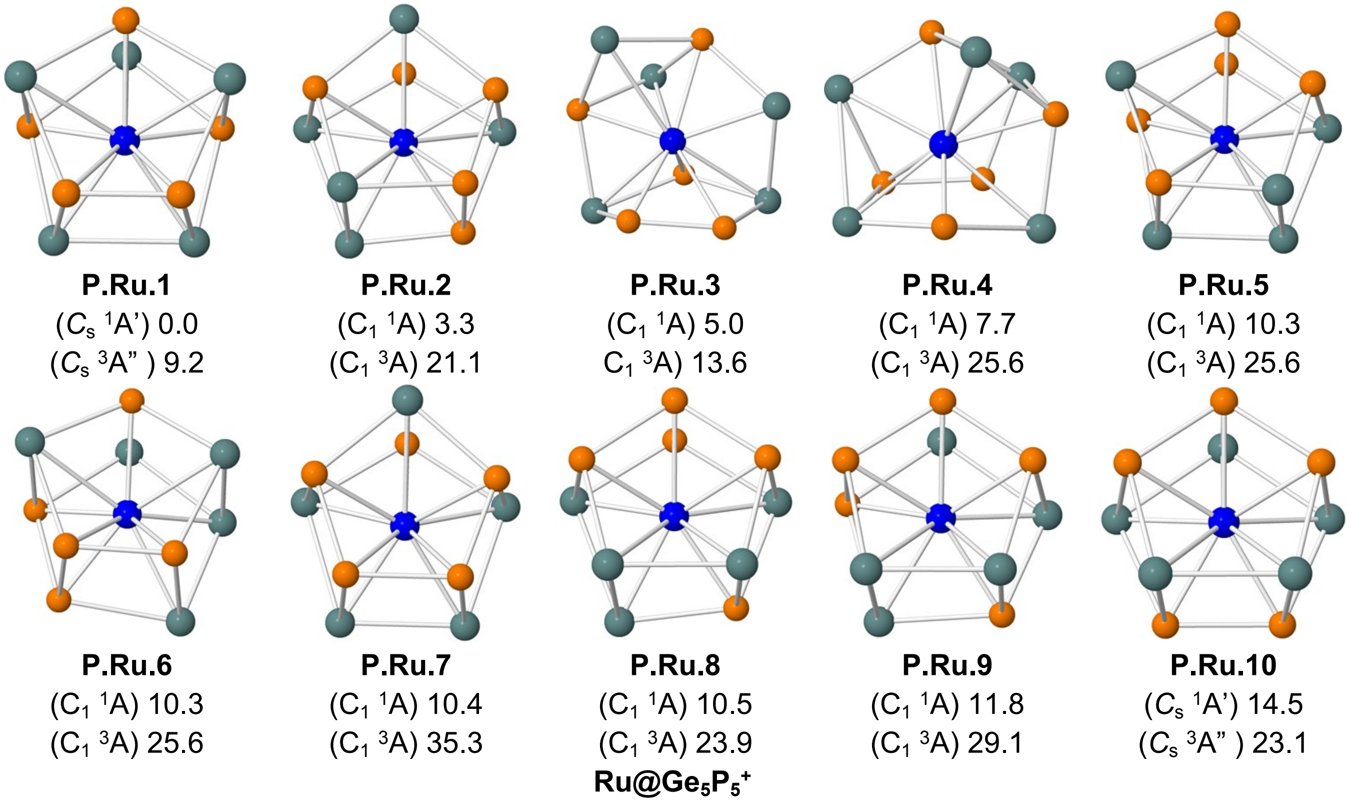

Figure S2. Shapes, and relative energies (in kcal/mol) of the lower-lying isomer of $RuGe_5P_5^+$ cluster. The geometry optimizations and energy calculations were performed using B3P86 functional with 6-311+G(d) basis set for Ge,P and aug-cc-pVTZ-PP basis set for Ru.

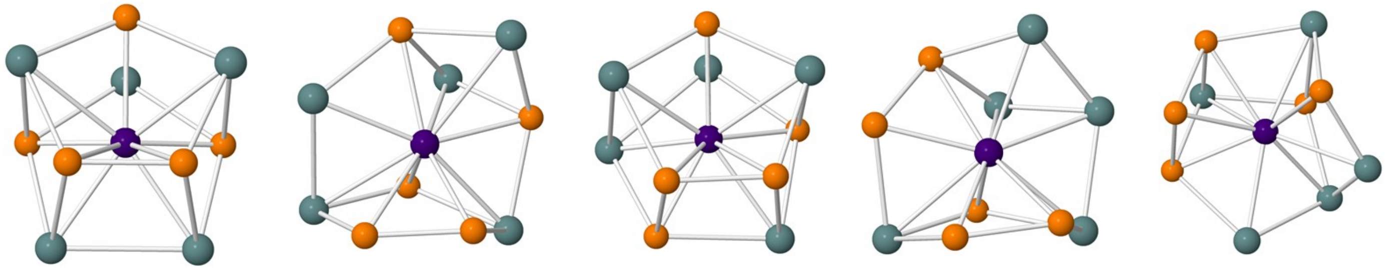

P.Os.1	P.Os.2	P.Os.3	P.Os.4	P.Os.5
(C_s ^{1}A’) 0.0	(C_1 ^{1}A) 2.4	(C_1 ^{1}A) 3.5	(C_1 ^{1}A) 5.4	(C_1 ^{1}A) 6.6
(C_s ^{3}A”) 16.5	(C_1 ^{3}A) 9.1	(C_1 ^{3}A) 21.**0**	(C_1 ^{3}A 18.3	(C_1 ^{3}A) 16.2

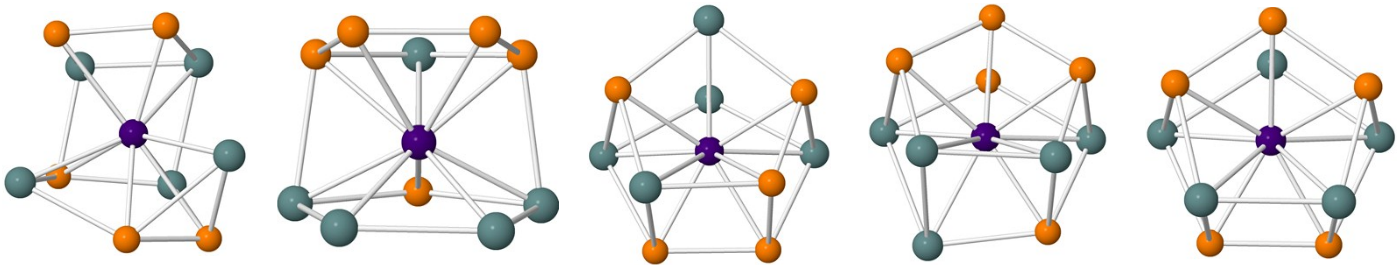

P.Os.6	P.Os.7	P.Os.8	P.Os.9	P.Os.10
(C_1 ^{1}A) 6.7	(C_1 ^{1}A) 9.3	(C_1 ^{1}A) 10.5	(C_1 ^{1}A) 11.0	(C_s ^{1}A’) 14.8
(C_1 ^{3}A) 12.7	(C_1 ^{3}A) 26.8	(C_1 ^{3}A) 24.8	(C_1 ^{3}A) 23.8	(C_s ^{3}A”) 29.0
		**Os@Ge5P5+**		

Figure S3. Shapes, and relative energies (in kcal/mol) of the lower-lying isomer of $OsGe_5P_5^+$ cluster. The geometry optimizations and energy calculations were performed using B3P86 functional with 6-311+G(d) basis set for Ge,P and aug-cc-pVTZ-PP basis set for Os.

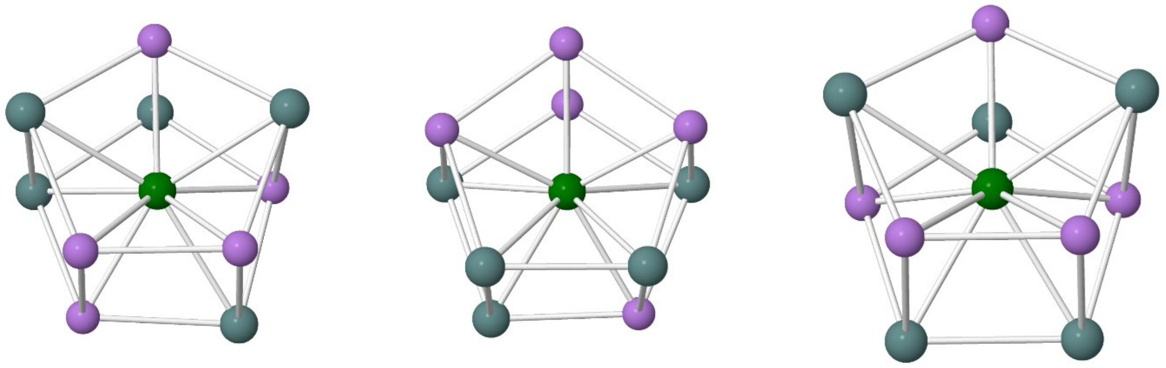

As.Fe.1 (${C_1}$ ^{1}A) 3.3 (${C_1}$ ^{3}A 0.0 (${C_1}$ ^{5}A) 40.6

As.Fe.2 (${C_1}$ ^{1}A) 8.6 (${C_1}$ ^{3}A) 0.9 (${C_1}$ ^{5}A) 45.0

As.Fe.3 (${C_s}$ ^{1}A') 1.1 (${C_s}$ ^{3}A')' 2.1 (${C_s}$ ^{5}A') 35.7

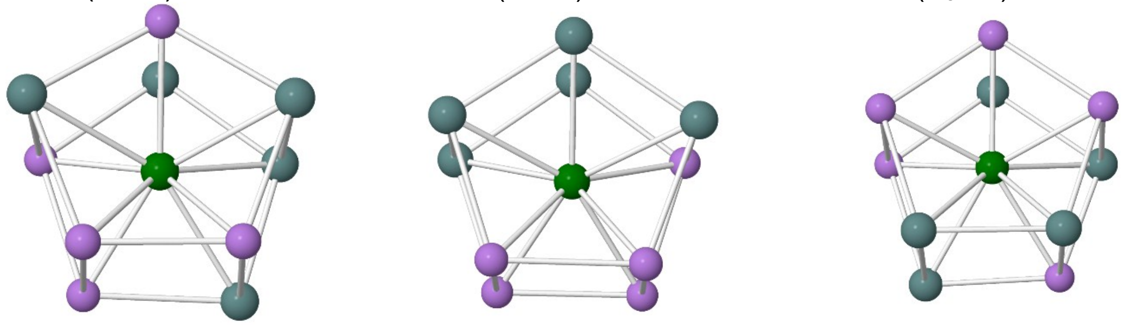

As.Fe.4 (${C_1}$ ^{1}A) 8.6 (${C_1}$ ^{3}A) 2.3 (${C_1}$ ^{5}A) 32.3

As.Fe.5 (${C_1}$ ^{1}A) 17.2 (${C_1}$ ^{3}A) 6.1 (${C_1}$ ^{5}A) 53.3

As.Fe.6 (${C_1}$ ^{1}A) 9.0 (${C_1}$ ^{3}A) 6.3 (${C_1}$ ^{4}A) 43.0

Figure S4. Shapes, and relative energies (in kcal/mol) of the lower-lying isomer of $FeGe_5As_5^+$ cluster. The geometry optimizations and energy calculations were performed using B3P86 functional with 6-311+G(d) basis set for Ge,As and aug-cc-pVTZ basis set for Fe.

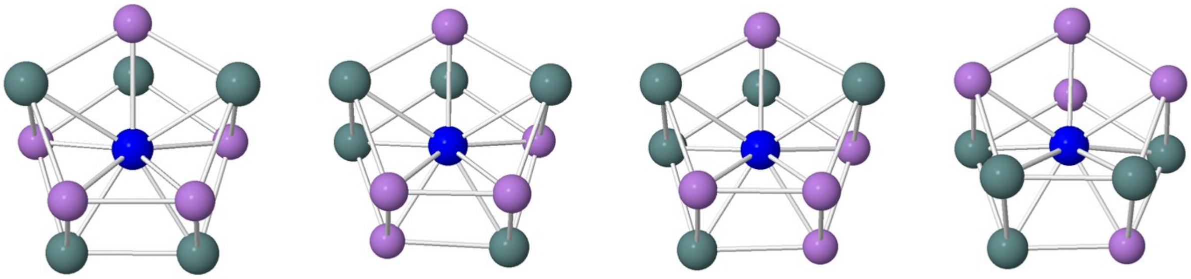

As.Ru.1 (${C_s}$ ^{1}A') 0.0

As.Ru.2 (${C_1}$ ^{1}A) 2.5

As.Ru.3 (${C_1}$ ^{1}A) 7.8

As.Ru.4 (${C_1}$ ^{1}A) 8.3

(C_s ^{3}A'') 17.8 | (C_1 ^{3}A) 17.4 | (C_1 ^{3}A) 20.8 | (C_1 ^{3}A) 19.6

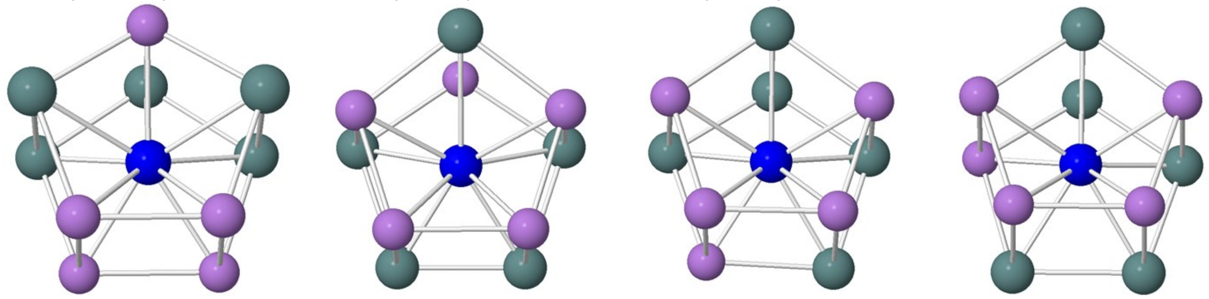

As.Ru.5 (C_s ^{1}A') 9.8 (C_s ^{3}A'') 25.2

As.Ru.6 (C_s ^{1}A') 10.2 (C_s ^{3}A'') 28.0

As.Ru.7 (C_1 ^{1}A) 10.7 (C_1 ^{3}A) 30.0

As.Ru.8 (C_1 ^{1}A) 13.0 (C_1 ^{3}A) 30.4

Figure S5. Shapes, and relative energies (in kcal/mol) of the lower-lying isomer of $RuGe_5As_5^+$ cluster. The geometry optimizations and energy calculations were performed using B3P86 functional with 6-311+G(d) basis set for Ge,As and aug-cc-pVTZ-PP basis set for Ru.

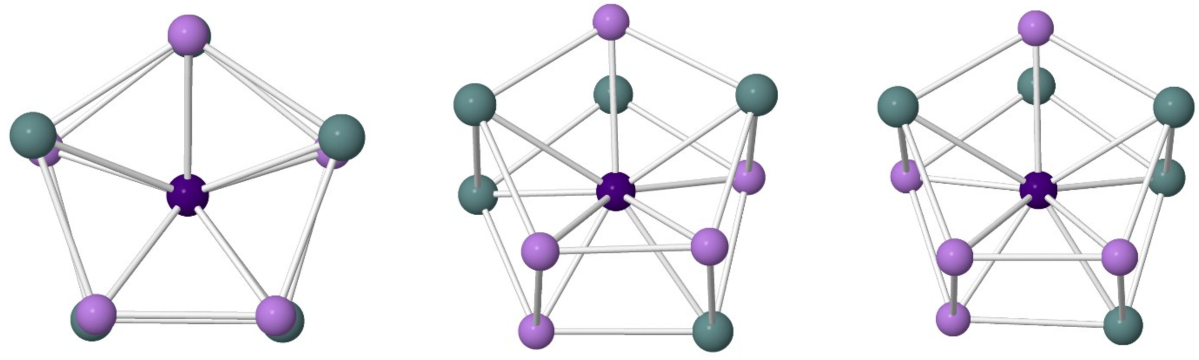

As.Os.1 (C_s ^{1}A') 0.0 (C_s ^{3}A'') 17.5

As.Os.2 (C_1 ^{1}A) 2.43 (C_1 ^{3}A) 17.5

As.Os.3 (C_1 ^{1}A) 7.8 (C_1 ^{3}A) 20.6

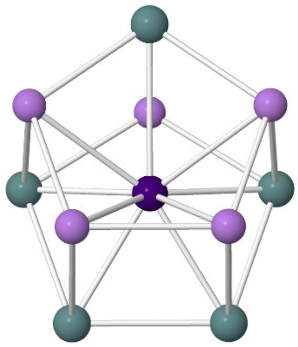

GeAs.Os.5
(C_1 ^{1}A) 9.7
(C_1 ^{3}A) 25.0

Figure S6. Shapes, and relative energies (in kcal/mol) of the lower-lying isomer of $OsGe_5As_5^+$ cluster. The geometry optimizations and energy calculations were performed using B3P86 functional with 6-311+G(d) basis setfor Ge,As and aug-cc-pVTZ basis set for Os.

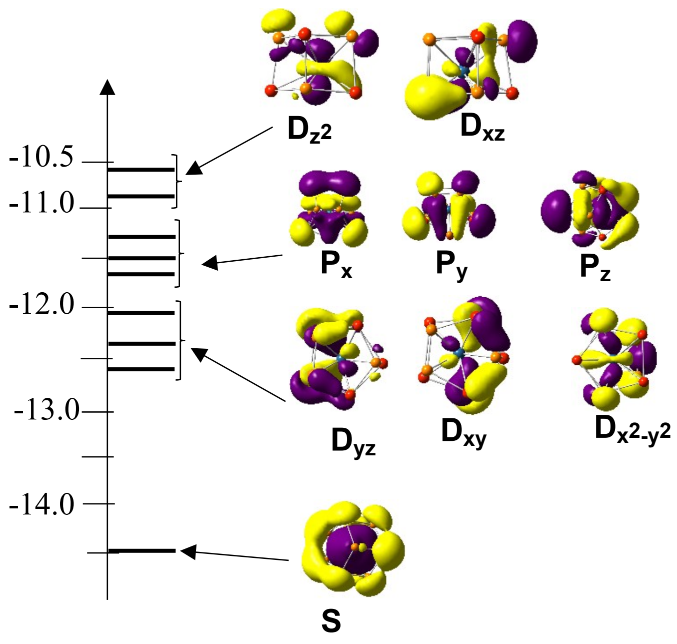

Figure S7. The MO diagram containing 18 electrons of $RuGe_5P_5^+$ structure.

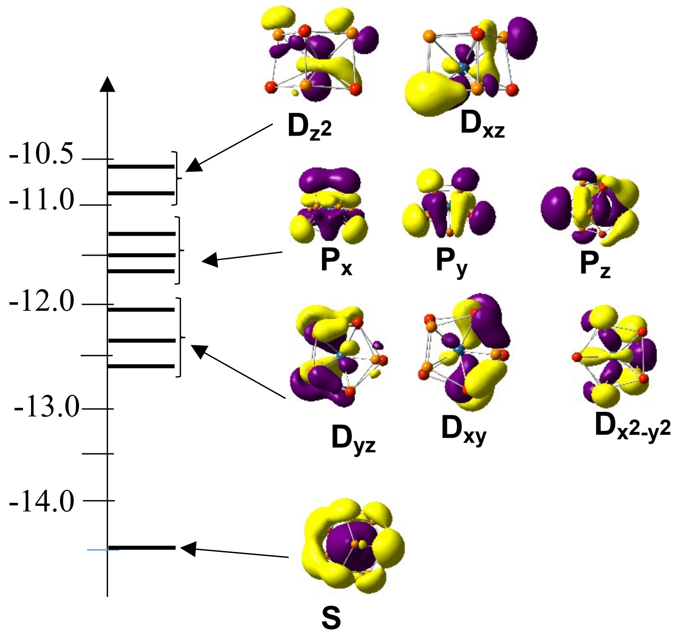

Figure S8. The MO diagram containing 18 electrons of $OsGe_5P_5^+$ structure.

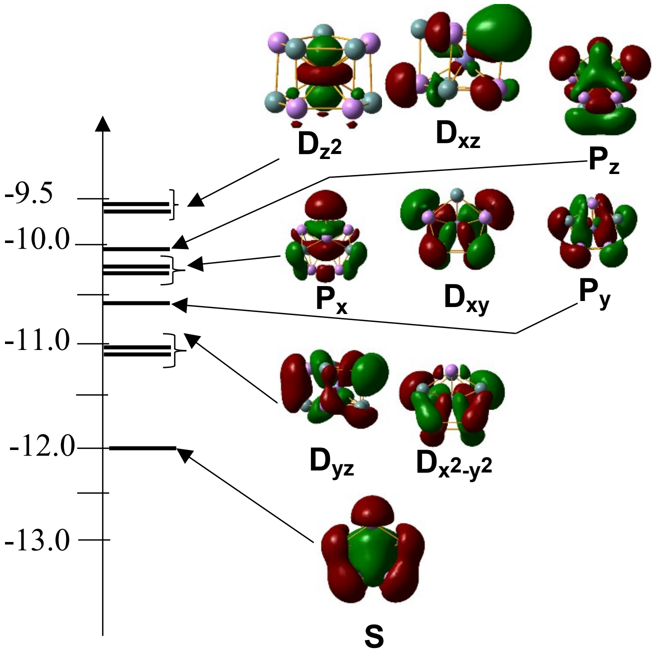

Figure S9. The MO diagram containing 18 electrons of $FeGe_5As_5^+$ structure.

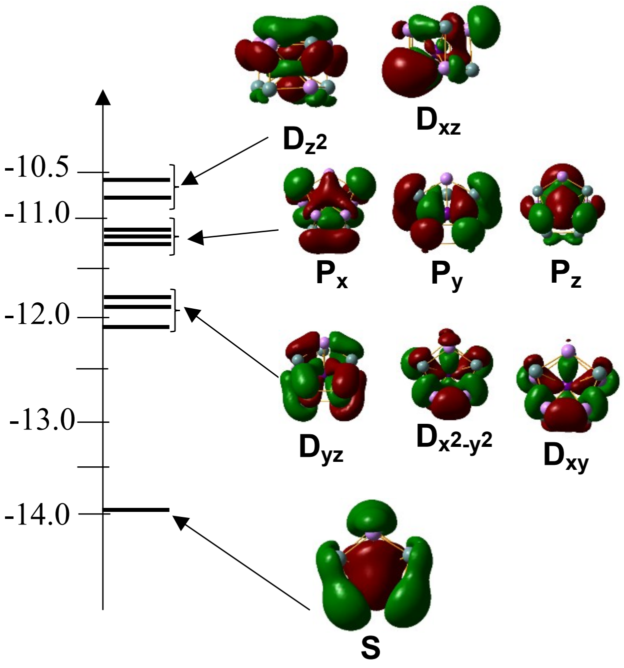

Figure S10. The MO diagram containing 18 electrons of $RuGe_5As_5^+$ structure.

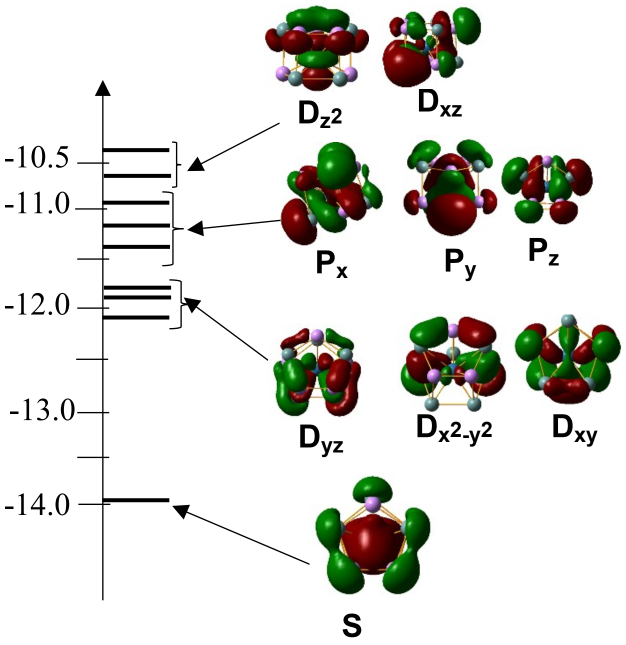

Figure S11. The MO diagram containing 18 electrons of $OsGe_5As_5^+$ structure.
